# Small-intestinal TG2-specific plasma cells at different stages of coeliac disease

**DOI:** 10.1186/s12865-018-0275-7

**Published:** 2018-12-06

**Authors:** Minna Hietikko, Outi Koskinen, Kalle Kurppa, Kaija Laurila, Päivi Saavalainen, Teea Salmi, Tuire Ilus, Heini Huhtala, Katri Kaukinen, Katri Lindfors

**Affiliations:** 1 Celiac Disease Research Center, Faculty of Medicine and Life Sciences, University of Tampere, P.O. Box 100, 33014 Tampere, Finland; 2 Tampere Center for Child Health Research, University of Tampere, Tampere, Finland; 3 Department of Paediatrics, Tampere University Hospital, Tampere, Finland; 4 Department of Medical and Clinical Genetics and the Research Programs Unit, Immunobiology, University of Helsinki, Helsinki, Finland; 5 Department of Dermatology, Tampere University Hospital, Tampere, Finland; 6 Department of Gastroenterology and Alimentary Tract Surgery, Tampere University Hospital, Tampere, Finland; 7 Faculty of Social Sciences, University of Tampere, Tampere, Finland; 8 Department of Internal Medicine, Tampere University Hospital, Tampere, Finland

**Keywords:** Coeliac disease, Gluten, Transglutaminase 2, Autoantibody, Small intestine

## Abstract

**Background:**

In coeliac disease, ingestion of gluten induces the production of transglutaminase 2 (TG2)-targeted autoantibodies by TG2-specific plasma cells present at high frequency in the small intestinal mucosa in untreated disease. During treatment with a gluten-free diet (GFD), the number of these cells decreases considerably. It has not been previously investigated whether the cells are also present prior to development of villous atrophy, or in non-responsive patients and those with dietary lapses. We aimed to define the frequency of small bowel mucosal TG2-specific plasma cells in coeliac disease patients with varying disease activity, and to investigate whether the frequency correlates with serum and small intestinal TG2-targeting antibodies as well as mucosal morphology and the number of intraepithelial lymphocytes.

**Results:**

Mucosal TG2-specific plasma cells were found in 79% of patients prior to development of mucosal damage, in all patients with villous atrophy, and in 63% of the patients after 1 year on GFD. In these disease stages, TG2-specific plasma cells accounted for median of 2.3, 4.3, and 0.7% of all mucosal plasma cells, respectively. After long-term treatment, the cells were present in 20% of the patients in clinical remission (median 0%) and in 60% of the patients with poor dietary adherence (median 5.8%). In patients with non-responsive coeliac disease despite strict GFD, the cells were found in only one (9%) subject; the cells accounted for 2.4% of all plasma cells. A positive correlation between the percentage of TG2-specific plasma cells and serum TG2 antibody levels (r_S_ = 0.69, *P* < 0.001) and the intensity of mucosal TG2-targeting IgA deposits (r_S_ = 0.43, P < 0.001) was observed.

**Conclusions:**

Our results show that TG2-specific plasma cells are already detectable prior to villous atrophy, and that generally their frequency increases during overt disease. By contrast, on GFD, the percentage of these cells decreases. Overall, the presence of TG2-specific plasma cells in the small bowel mucosa mirrors the presence of gluten in the diet, but the frequency is not always parallel to the level of serum or intestinal TG2 antibodies. These findings increase the knowledge about the development of the TG2 plasma cell responses especially in the early phases of coeliac disease.

**Electronic supplementary material:**

The online version of this article (10.1186/s12865-018-0275-7) contains supplementary material, which is available to authorized users.

## Background

In coeliac disease, dietary gluten in wheat, rye, and barley functions as a driving antigen for an abnormal immune response that develops in genetically susceptible individuals carrying the human leukocyte antigen (HLA)-DQ2 or -DQ8 haplotypes. The disease is characterised by small-bowel mucosal damage which develops gradually from normal villous morphology to inflammation and finally to villous atrophy with crypt hyperplasia diagnostic of coeliac disease. The intestinal damage is often coupled with numerous gastrointestinal symptoms, although various extraintestinal manifestations are also prevalent. A specific characteristic of coeliac disease is the generation of immunoglobulin class A (IgA) antibodies towards the main autoantigen, transglutaminase 2 (TG2) [1]. These autoantibodies are generally found in the circulation of coeliac disease patients [2] and as deposits in the small intestinal mucosa below the subepithelial basement membrane and around blood vessels [3]. Interestingly, intestinal TG2-targeted deposits can be detected even prior to manifest mucosal damage and in the absence of serum antibodies [4–6]. Upon removal of gluten from the diet, the only currently available treatment, the clinical symptoms and histopathological changes in the small intestine resolve, and both the circulating and intestinal antibodies disappear within 1 year in most patients [7]. However, a subset of patients fails to respond to the dietary treatment and the villous atrophy persists despite a strict gluten-free diet (GFD). The most common reason for persistent villous atrophy is either advertent or inadvertent gluten intake or, in rare cases, refractory coeliac disease [8].

TG2-targeting antibodies were long thought to be generated by intestinal plasma cells [9–11], but recent data suggests that they might also be produced in lymphoid tissues outside the gut [12]. In the small intestine, the TG2-specific plasma cells are present at high frequency during the active disease [10, 11], and they decrease considerably within 6–12 months after commencement of a strict GFD [11]. However, no data exist regarding the presence of these cells in the early phases of coeliac disease when the mucosal morphology is still normal. In addition, their existence in non-responding coeliac disease patients or those with dietary lapses has not been previously investigated. With this in mind, we enumerated the TG2-specific plasma cells in untreated and treated coeliac patients with varying degrees of disease activity, and investigated whether the number of these cells correlates with serum TG2 antibody levels, the intensity of mucosal TG2-targeting IgA deposits, and small intestinal mucosal morphology and inflammation.

## Methods

### Patients and study design

The study cohort comprised 46 coeliac disease patients who underwent upper gastrointestinal endoscopy at the Department of Gastroenterology and Alimentary Tract Surgery of Tampere University Hospital (Table 1). Fifteen of the patients were clinically suspected of having coeliac disease based on gastrointestinal symptoms and positive coeliac disease-specific autoantibodies (endomysial and/or TG2 antibodies) despite having normal small bowel mucosa (villous height crypt depth ratio (Vh/CrD) ≥ 2). These patients were prospectively followed up while they continued on a normal gluten-containing diet for 1 year, during which villous atrophy developed (Vh/CrD < 2) in all patients. Thereafter, the patients started a GFD, and after 1 year on the diet, their mucosal morphology had recovered. Small bowel samples from 14 of the patients at the time of the normal mucosal morphology, all 15 patients at the time of the villous atrophy, and 11 of the patients after 1 year on a GFD were available for the current study (Table 1).

**Table 1:** Demographic data and the small-bowel mucosal and serological findings of patients participating in the study

	Prospectively studied coeliac patients (*n* = 15)			Long-term treated coeliac patients (*n* = 31)			Disease controls (*n* = 25)	
	CD prior to atrophy *n* = 14	Overt CD *n* = 15	1 year GFD *n* = 11	Patients in remission *n* = 15	Non-responding CD *n* = 11	Patients with dietary lapses *n* = 5	Gluten sensitivity *n* = 18	Dyspepsia *n* = 7
Female; n (%)	10 (71)	10 (67)	7 (64)	10 (67)	7 (64)	5 (100)	16 (89)	1 (14)
Age; median (range), years	55 (16–70)	55 (17–71)	57 (28–72)	59 (24–66)	49 (40–76)	51 (31–77)	49 (24–65)	47 (24–76)
Duration of GFD; median (range), years	0	0	1 (1–1)	8 (3–34)	7 (3–24)	10 (9–17)	0	0
HLA-DQ2 or -DQ8-positive; n (%)	14 (100)	15 (100)	11 (100)	15 (100)	11 (100)	4^a^ (100)	9 (50)	1 (14)
EmA; median (range), titer	1:100 (0–1:2000)	1:100 (0–1:4000)	0 (0–1:50)	0 (0)	0 (0–1:5)	1:200 (0–1:2000)	0 (0)	0 (0)
TG2 abs; median (range), U/ml	9.4 (3.3- > 100)	11.9 (4.2- > 100)	3.9 (0–8.6)	0.5 (0–2.8)	1.3 (0–9.9)	56.9 (12.9- > 100)	1.4 (0–4.1)	0.6 (0–2.8)
Mucosal TG2-IgA deposits present; n (%)	14 (100)	14^a^ (100)	7^b^ (88)	5 (33)	10^a^ (100)	3^c^ (100)	4 (22)	0 (100)
Vh/CrD; mean (95% CI), ratio	2.9 (2.6–3.1)	1.4 (1.1–1.7)	3.4 (2.4–4.5)	3.4 (3.2–3.6)	0.2 (0.0–0.4)	0.5 (−0.3–1.3)	3.5 (3.0–4.1)	3.5 (3.0–4.1)
CD3^+^ IELs; median (range), cells/mm	54 (12–79)	67 (38–116)	39 (23–80)	42 (25–77)	60 (30–109)	50 (38–69)	21 (7–59)	26 (16–40)
αβ^+^ IELs; median (range), cells/mm	30 (12–50)	43 (21–75)	22 (14–43)	31 (20–46)	44 (26–105)	38 (35–56)	16 (5–26)	22 (17–31)
γδ^+^ IELs; median (range), cells/mm	18.8 (0–38.5)	23.7 (14–58.7)	v13.6 (1.4–56.3)	14.0 (7.3–27.8)	12.1 (0–37.8)	13.0 (4.4–20.5)	2.7 (0–10.2)	1.6 (0.7–16.1)

*abs* antibodies, *CD* coeliac disease, *CI* confidence interval, *EmA* endomysial antibodies, *GFD* gluten-free diet, *GS* gluten sensitive; *IgA* immunoglobulin A; *IELs* intraepithelial lymphocytes; TG2-abs, transglutaminase 2 antibodies; *Vh/CrD* villous height crypt depth ratio

Reference values set at 2.0 for Vh/CrD, 37 cells/mm for CD3^+^ IELs, 25 cells/mm for αβ^+^ IELs, and 4.3 cell/mm for γδ+ IELs (Järvinen et al., [15])

Cut-off value for TG2 antibodies ≥ 5 AU/ml

^a^Data missing from one patient

^b^Data missing from three patients

^c^Data missing from two patients

Furthermore, 15 coeliac disease patients on a long-term GFD without symptoms and evincing full histological recovery, 11 non-responsive coeliac disease patients with persistent villous atrophy despite a strict GFD, and five patients with poor dietary adherence were investigated. Twenty patients with self-reported gluten sensitivity experiencing abdominal symptoms after consumption of gluten-containing products [13] and seven patients with dyspepsia served as the non-coeliac controls in the study. All controls had been excluded for coeliac disease, as demonstrated by negative serology and normal small bowel mucosal morphology. The demographic data and small-bowel mucosal and serological findings of all subjects are reported in Table 1.

The study protocol was approved by the Ethics Committee of the Pirkanmaa Hospital District, Tampere, Finland, and written informed consent was obtained from all participating subjects.

### Small-intestinal mucosal morphology and inflammation

Small-intestinal mucosal biopsies were obtained upon upper gastrointestinal endoscopy. For morphological studies, one formalin-fixed biopsy sample was stained with haematoxylin and eosin to determine the villous height-crypt depth ratios (Vh/CrD) according to a previously published procedure [14]. A ratio of ≥2 was considered normal. One of the biopsies was submerged in optimal cutting temperature compound (OCT; Tissue-Tek, Sakura Finetek Europe, Holland), followed by snap-freezing in liquid nitrogen. Thereafter, 5-μm-thick sections were cut. According to an established protocol [15], the sections were stained for CD3^+^, αβ^+^, and γδ^+^ intraepithelial lymphocyte (IEL) subsets. The reference values were 37 cells/mm, 25 cells/mm, and 4.3 cells/mm for CD3^+^ IELs, γδ^+^ IELs and αβ^+^ IELs, respectively [15].

### Serological measurements and HLA genotyping

Serum endomysial antibodies (EmA) in IgA class were determined by an indirect immunofluorescence method exploiting human umbilical cord as substrate. A dilution of 1:≥5 was considered positive [16]. Serum IgA-class TG2 antibodies were measured by a commercially available enzyme-linked immunosorbent assay (Celikey®, Phadia, Freiburg, Germany) in samples diluted 1:100. A titre of ≥ 5 AU/ml was set as the cut-off for positivity.

SSP DQB1 low-resolution kit (Olerup SSP AB, Saltsjöbaden, Sweden), DELFIA Celiac Disease Hybridization Assay (PerkinElmer Life and Analytical Sciences, Wallac Oy, Turku, Finland) or HLA-tagging single-nucleotide peptides [17] were used for HLA genotyping.

### Small-intestinal TG2-specific IgA deposits

For the determination of mucosal TG2-targeting IgA deposits, frozen sections were stained with mouse monoclonal anti-TG2 antibody (CUB7402; NeoMarkers, Fremont, California, USA), followed by detection with fluorescein isothiocyanate (FITC) -labelled rabbit anti-human IgA antibody (Dako A/S, Glostrup, Denmark) [3]. Based on their intensity along the basement membrane in the villous-crypt area, the deposits were graded blinded as a negative, or a weak, moderate, or strong positive, as described previously [6].

### Small-intestinal TG2-specific plasma cells

An earlier described technique was used to detect mucosal TG2-specific plasma cells [10]. Initially, 5-μm-thick frozen sections were air-dried for 20 min at room temperature (RT). After washing in PBS, the sections were incubated with biotinylated human recombinant TG2 (2 μg/ml; T002, Zedira) for 45 min at RT. Biotinylation was performed using EZ-Link® Sulfo-NHS-LC-Biotin (Thermo Scientific, Waltham, MA, USA) according to the instructions provided by the manufacturer. Thereafter, the sections were incubated with rhodamine-labelled streptavidin (1:1000; KPL, Gaithersburg, MD, USA) for 30 min at RT. Plasma cells were identified using a mouse monoclonal CD138 antibody (1:25; B-A38, Bio-Rad), followed by goat anti-mouse IgG Alexa Fluor 488 (1:2000; A-11001, Thermo-Fisher Scientific). Stainings were analysed at 20x and 40x magnification (Olympus BX60F5, Olympus Optical Co. LTD, Japan) on two consecutive small intestinal biopsy sections and the percentage of TG2-specific cells out of all *lamina propria* plasma cells in the entire section was determined.

### Statistical analyses

Data are expressed as the number of subjects (n) and percentages, or as medians and ranges. Statistical analyses were performed using the Wilcoxon test or Mann–Whitney test as appropriate. Correlation was evaluated using Spearman’s correlation. Statistical testing was performed using statistical analysis software (IBM SPSS Statistics, SPSS Inc., Chicago, IL, USA). A *P*-value < 0.05 was considered statistically significant.

## Results

**Fig. 1:**
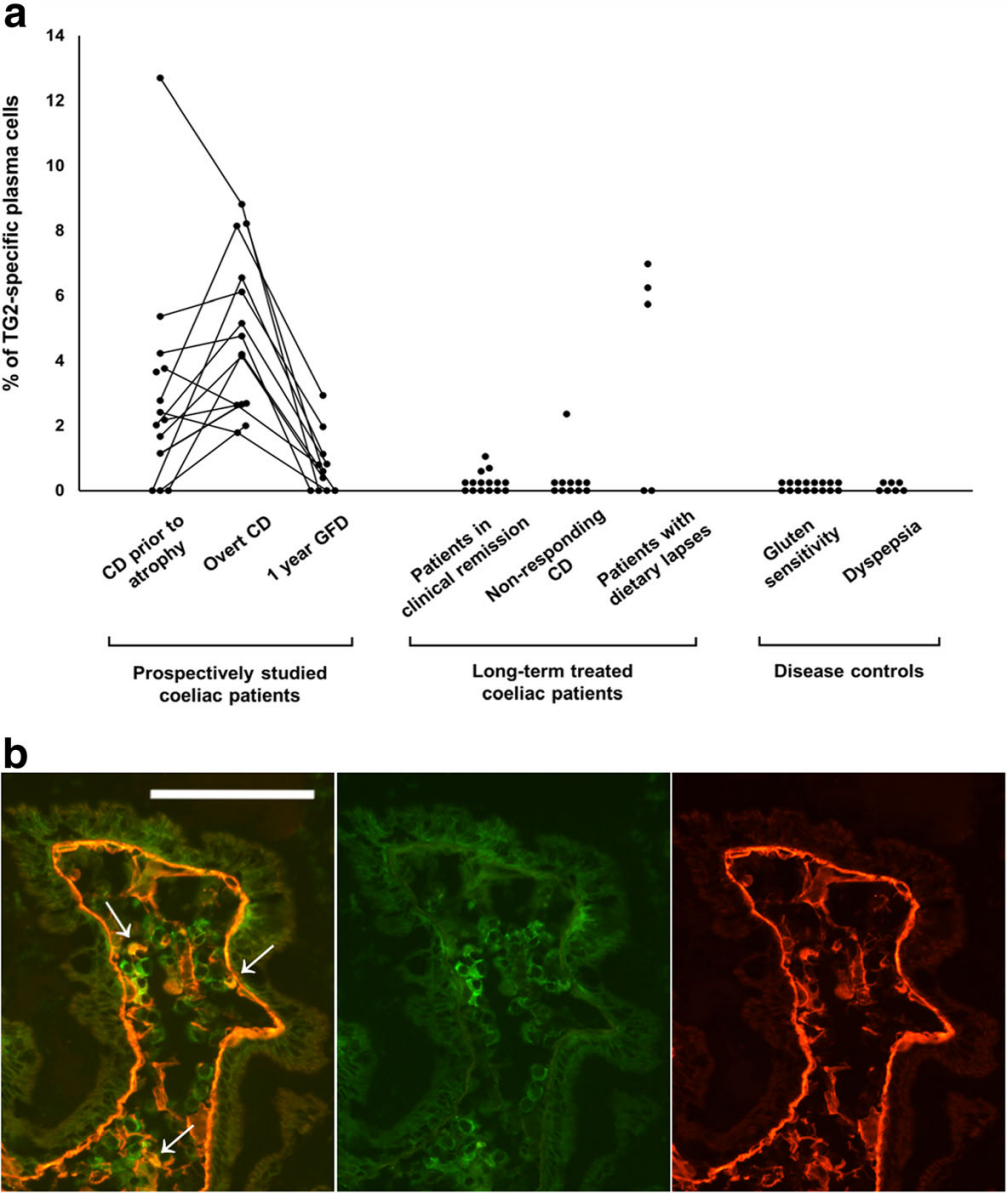
**a.** The percentage of small-bowel mucosal transglutaminase 2 (TG2) -specific plasma cells out of all *lamina propri*a plasma cells in the different patient groups. **b.** Immunofluorescence staining for transglutaminase 2 (TG2) antibodies and plasma cells in small-bowel mucosal sections. Representative picture of a coeliac disease patient prior to villous atrophy showing positive staining for TG2-specific plasma cells (arrows). Recombinant TG2 (red), plasma cell marker CD138 (green), and their colocalisation (yellow) at 20x magnification. Scale bar = 100 μm. Abbreviations: CD, coeliac disease; GFD, gluten-free diet

Of the 15 prospectively studied coeliac disease patients, TG2-specific plasma cells were already present in 11 out of the 14 available small bowel samples (79%) from the patients before the development of villous atrophy. The median percentage of the cells was 2.3% (range 0–12.7%) of all *lamina propria* plasma cells (Fig. 1a and b). After continuing on a gluten-containing diet for 1 year and developing overt small bowel mucosal damage, all fifteen patients had intestinal TG2-specific plasma cells, and the median percentage of the cells was 4.3% (range 1.8–8.8%) (*P* = 0.055 when compared to patients with early-stage coeliac disease). By contrast, after 1 year on a GFD, the cells were found in 7 out of 11 (64%) patients with available samples, and the median percentage of the cells significantly decreased to 0.7% (range 0–2.9%, *P* = 0.003) when compared to the overt disease.

In long-term GFD-treated patients in clinical remission responding well to dietary treatment, only a few remaining TG2-specific plasma cells (median 0.0%, range 0–1.1%) were detected in 3 out of 15 (20%) of the patients (Fig. 1a and b). In non-responding coeliac disease patients on a strict GFD, the cells were mostly absent, being present in only one patient (9%) who had 2.4% of TG2-specific cells out of all *lamina propria* plasma cells. Of the coeliac disease patients with dietary lapses, three out of five (60%) had TG2-specific plasma cells, the median being 5.8% (range 0–7.0%). No TG2-specific plasma cells were found in any of the non-coeliac control patients with either gluten sensitivity or dyspepsia.

**Fig. 2:**
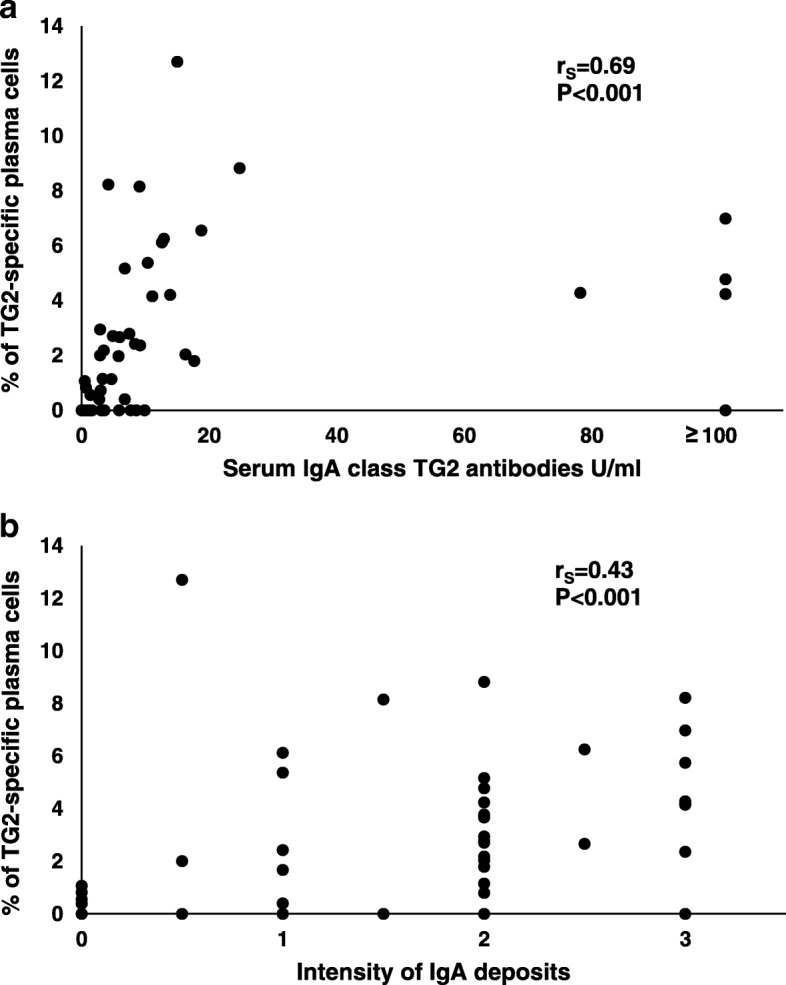
Correlation between the percentage of small intestinal transglutaminase 2 (TG2) -specific plasma cells and serum IgA class TG2 antibody levels (**a**) and the intensity of small intestinal TG2-targeting immunoglobulin a (IgA) deposits (**b**) in all coeliac disease patients. Grading of IgA deposits as follows: 0 = negative, 1 = weak, 2 = moderate, 3 = strong

A positive correlation between the percentage of TG2-specific plasma cells and serum TG2 antibody levels (r_S_ = 0.690, *P* < 0.001, Fig. 2a) as well as EmA (r_S_ = 0.712, P < 0.001) was observed when data from all coeliac disease patient groups were included in the analysis. Similarly, the percentage of the TG2-specific plasma cells correlated with the intensity of the small intestinal IgA deposits in all coeliac disease patients (r_S_ = 0.430, *P* < 0.001) (Fig. 2b). However, the percentage of TG2-specific plasma cells did not correlate with serum or mucosal TG2 antibodies in any of the individual coeliac disease patient groups (Additional file 1 Table S1). Considering all coeliac disease patients, there was no correlation between the percentage of the plasma cells and Vh/CrD. Of the IELs, there was a modest correlation between the percentage of TG2-specific plasma cells and γδ^+^ IELs. Detailed information about the correlations is presented in Additional file 1 Table S1.

## Discussion

In the current study, we have discovered that TG2-specific plasma cells are already present in most patients prior to detectable mucosal damage. Moreover, we showed that in the majority of cases the percentage of these cells increases upon continuous gluten intake and the subsequent development of villous atrophy. On a strict GFD, the percentage of these cells declines. After long-term treatment, the cells are mostly absent both in patients in clinical remission and in non-responding patients with persistent villous atrophy. By contrast, TG2-specific plasma cells can be detected in patients with dietary lapses. In controls, even those with gluten-related symptoms, no cells were detected.

Our results from untreated and treated coeliac disease patients are in line with previous studies [10, 11], showing that the amount of TG2-specific plasma cells is elevated in the small intestinal mucosa at the time of diagnosis and this amount decreases on a GFD. It has earlier been shown that the percentage of these cells out of all *lamina propria* plasma cells accounts for up to 24% in overt disease, being on average 10% [10, 11]. However, in the current study, the corresponding percentages were lower. Our patients had been recruited to the study while still having normal mucosal morphology, and they developed overt villous atrophy within a one-year follow-up on a gluten-containing diet. Thus, it is conceivable that the patients had had flat mucosal lesion for a reasonably shorter time than in the previous studies, which might explain the lower percentages of TG2-specific plasma cells in our study.

TG2-specific plasma cells correlated positively with serum TG2 antibody levels when the data of all coeliac disease patients were analysed together. This correlation most likely mirrors the responsiveness of the plasma cells to gluten exposure which is not surprising in the light that TG2 antibodies have been suggested to arise by a hapten-carrier-like mechanism involving TG2-catalysed generation of gluten-TG2 complexes [18, 19]. On the other hand, correlations between the percentages of the plasma cells and serum antibody levels were not detected when different coeliac disease groups were analysed separately; this is in agreement with previous findings in untreated coeliac disease patients [11]. It has been proposed that the lack of correlation could be explained by the production of the antibodies also outside the gut [11]. This concept has recently been further supported by the finding that coeliac patient serum and intestinal TG2 antibodies are clonally related but have different molecular compositions, pointing to different sites of origin [12]. Such extraintestinal production of TG2 antibodies could also explain why some patients in our study had serum TG2 antibodies in the absence of intestinal TG2-specific plasma cells.

Although small intestinal TG2-specific plasma cells are not likely to be the major source of serum TG2 antibodies [12], it would be logical to assume that the TG2 antibodies bound to their antigen in the small intestinal mucosa and predicting forthcoming mucosal damage are produced locally by *lamina propria* TG2-specific plasma cells. In this study, we observed a correlation between the percentage of the plasma cells and the intensity of mucosal TG2-specific IgA deposits, which supports this hypothesis. Interestingly, however, TG2-specific plasma cells were mostly absent in non-responding coeliac patients, even though they all presented with strong TG2-targeting IgA deposits in the small intestinal mucosa despite a strict GFD. It has been proposed that the long persistence of small intestinal IgA deposits in non-responsive patients on a strict diet may be explained, for instance, by the high avidity binding of the IgA antibodies to small intestinal TG2 [6, 7]. This could explain the presence of IgA deposits in the absence of TG2 antibody-secreting plasma cells in our non-responsive patients. However, it does not provide an explanation for the presence of IgA deposits in the absence of plasma cells in the small subset of patients prior to development of villous atrophy. Similarly, it does not explain the presence of weak IgA deposits without TG2-antibody secreting plasma cells in a few gluten-sensitive control patients. Whether extraintestinal production of TG2 antibodies occurring for instance in the bone marrow, spleen and lymph nodes contributes to the appearance of mucosal IgA deposits remains to be addressed in future studies.

## Conclusions

We conclude that the TG2-specific plasma cells are already present in the early phases of coeliac disease when the mucosal morphology is still normal, their percentage increases upon the development of villous atrophy and decreases on a GFD. Overall, the frequency of TG2 antibody-secreting plasma cells in the different phases of coeliac disease reflects the presence of gluten in the diet, but the frequency of these cells is not always parallel with serum TG2 antibody levels or the intensity of small intestinal TG2-targeting IgA deposits. Our findings widen the understanding of small-bowel mucosal TG2-specific plasma cells in coeliac disease and thus provide further insight into the generation of TG2 antibody responses.

## Additional file

Additional file 1: **Table S1.** Correlations between the percentage of TG2-specific plasma cells and other study parameters. (DOCX 15 kb)

## Abbreviations

EmA: Endomysial antibody
GFD: Gluten-free diet
HLA: Human leukocyte antigen
IEL: Intraepithelial lymphocyte
IgA: Immunoglobulin A
RT: Room temperature
TG2: Transglutaminase 2
Vh/CrD: Villous height-crypt depth ratio

## References

[1] Dieterich W, Ehnis T, Bauer M, Donner P, Volta U, Riecken E (1997). Identification of tissue transglutaminase as the autoantigen of celiac disease. Nat Med.

[2] Sulkanen S, Halttunen T, Laurila K, Kolho K, Korponay-Szabó IR, Sarnesto A (1998). Tissue transglutaminase autoantibody enzyme-linked immunosorbent assay in detecting celiac disease. Gastroenterology.

[3] Korponay-Szabo IR, Halttunen T, Szalai Z, Laurila K, Kiraly R, Kovacs JB (2004). In vivo targeting of intestinal and extraintestinal transglutaminase 2 by coeliac autoantibodies. Gut.

[4] Kaukinen K, Peräaho M, Collin P, Partanen J, Woolley N, Kaartinen T (2005). Small-bowel mucosal transglutaminase 2-specific IgA deposits in coeliac disease without villous atrophy: a prospective and randomized clinical study. Scand J Gastroenterol.

[5] Salmi T, Collin P, Järvinen O, Haimila K, Partanen J, Laurila K (2006). Immunoglobulin a autoantibodies against transglutaminase 2 in the small intestinal mucosa predict forthcoming coeliac disease. Aliment Pharmacol Ther.

[6] Salmi TT, Collin P, Korponay-Szabo IR, Laurila K, Partanen J, Huhtala H (2006). Endomysial antibody-negative coeliac disease: clinical characteristics and intestinal autoantibody deposits. Gut.

[7] Koskinen O, Collin P, Lindfors K, Laurila K, Maki M, Kaukinen K (2010). Usefulness of small-bowel mucosal transglutaminase-2 specific autoantibody deposits in the diagnosis and follow-up of celiac disease. J Clin Gastroenterol.

[8] Ilus T, Kaukinen K, Virta L, Huhtala H, Mäki M, Kurppa K (2014). Refractory coeliac disease in a country with a high prevalence of clinically-diagnosed coeliac disease. Aliment Pharmacol Ther.

[9] Marzari R, Sblattero D, Florian F, Tongiorgi E (2001). Not T, Tommasini a , et al. molecular dissection of the tissue transglutaminase antoantibody response in celiac disease. J Immunol.

[10] Di Niro R, Mesin L, Zheng N, Stamnaes J, Morrissey M, Lee J (2012). High abundance of plasma cells secreting transglutaminase 2-specific IgA autoantibodies with limited somatic hypermutation in celiac disease intestinal lesions. Nat Med.

[11] Di Niro R, Snir O, Kaukinen K, Yaari G, Lundin K, Gupta N, et al. Responsive population dynamics and wide seeding into the duodenal lamina propria of transglutaminase-2-specific plasma cells in celiac disease. Mucosal Immunol. 2016;9:254–64. 10.1038/mi.2015.57 PMC4703456 26153762

[12] Iversen R, Snir O, Stensland M, Kroll JE, Steinsbø Ø, Korponay-Szabó IR (2017). Strong clonal relatedness between serum and gut IgA despite different plasma cell origins. Cell Rep.

[13] Kaukinen K, Turjanmaa K, Mäki M, Partanen J, Venäläinen R, Reunala T (2000). Intolerance to cereals is not specific for coeliac disease. Scand J Gastroenterol.

[14] Taavela J, Koskinen O, Huhtala H, Lähdeaho M, Popp A, Laurila K (2013). Validation of morphometric analyses of small-intestinal biopsy readouts in celiac disease. PLoS One.

[15] Järvinen TT, Kaukinen K, Laurila K, Kyrönpalo S, Rasmussen M, Mäki M (2003). Intraepithelial lymphocytes in celiac disease. Am J Gastroenterol.

[16] Sulkanen S, Collin P, Laurila K, Mäki M (1998). IgA-and IgG-class antihuman umbilical cord antibody tests in adult coeliac disease. Scand J Gastroenterol.

[17] Koskinen L, Romanos J, Kaukinen K, Mustalahti K, Korponay-Szabo I, Barisani D (2009). Cost-effective HLA typing with tagging SNPs predicts celiac disease risk haplotypes in the Finnish, Hungarian, and Italian populations. Immunogenetics.

[18] Sollid LM, Molberg O, McAdam S, Lundin KE (1997). Autoantibodies in coeliac disease: tissue transglutaminase-guilt by association?. Gut.

[19] Stamnaes J, Sollid LM (2015). Celiac disease: autoimmunity in response to food antigen. Semin Immunol.

