# Supplementary material for: Small-intestinal TG2-specific plasma cells at different stages of coeliac disease
Source: BMC Immunol. 2018 Dec 6;19:36. doi: 10.1186/s12865-018-0275-7 (PMC6282384; doi:10.1186/s12865-018-0275-7)
Supplement: Supplementary file 1 — Table S1. Correlations between the percentage of TG2-specific plasma cells and other study parameters. (DOCX 15 kb) [file 12865_2018_275_MOESM1_ESM.docx]

|  |  | **TG2 abs** | **EmA** | **Mucosal TG2-IgA deposits** | **Vh/CrD** | **CD3^+^ IELs** | **αβ^+^ IELs** | **γδ^+^ IELs** |
| --- | --- | --- | --- | --- | --- | --- | --- | --- |
| **All CD patients** | | r_S_=0.690  P<0.001 | r_S_=0.712  P<0.001 | r_S_=0.430  P<0.001 | r_S_=-0.189 P=0.115 | r_S_=0.211  P=0.082 | r_S_=0.141  P=0.248 | r_S_=0.270  P=0.025 |
| **Prospectively studied**  **coeliac patients** | **CD prior to atrophy** | r_S_=0.235  P=0.514 | r_S_=-0.057  P=0.847 | r_S_=-0.153  P=0.602 | r_S_=0.215  P=0.461 | r_S_=0.394  P=0.182 | r_S_=-0.058  P=0.851 | r_S_=0.594  P=0.032 |
|  | **Overt CD** | r_S_=0.231  P=0.427 | r_S_=0.490  P=0.064 | r_S_=-0.62  P=0.833 | r_S_=0.377  P=0.165 | r_S_=-0.253  P=0.383 | r_S_=-0.244  P=0.401 | r_S_=-0.112  P=0.703 |
|  | **1 year GFD** | r_S_=-0.125  P=0.731 | r_S_=-0.300  P=0.931 | r_S_=0.262  P=0.531 | r_S_=-0.240  P=0.477 | r_S_=-0.395  P=0.229 | r_S_=-0.206  P=0.544 | r_S_=-0.284  P=0.398 |
| **Long-term treated coeliac patients** | **Patients in clinical remission** | r_S_=0.428  P=0.112 | ND | r_S_=-0.346  P=0.206 | r_S_=-0.164  P=0.559 | r_S_=0.064  P=0.821 | r_S_=0.335  P=0.222 | r_S_=0.250  P=0.368 |
|  | **Non-responding CD** | r_S_=0.401  P=0.222 | ND | r_S_=0.441  P=0.202 | r_S_=-0.191  P=0.573 | ND | r_S_=0.100  P=0.770 | ND |
|  | **Patients with dietary lapses** | ND | r_S_=0.872  P=0.054 | r_S_=-0.258  P=0.742 | r_S_=-0.287  P=0.640 | r_S_=0.154  P=0.805 | r_S_=0.108  P=0.863 | r_S_=0.564  P=0.322 |

**Supplementary table 1. Correlations between the percentage of TG2-specific plasma cells and other study parameters.**

ND not determined because of either non-sufficient number of samples or zero or low variance.

Abbreviations: abs; antibodies; CD, coeliac disease; EmA, endomysial antibodies; GFD, gluten-free diet; IgA, immunoglobulin A; IELs, intraepithelial lymphocytes; TG2, transglutaminase 2; Vh/CrD, villous height crypt depth ratio
